# TNF autovaccination induces self anti-TNF antibodies and inhibits metastasis in a murine melanoma model

**DOI:** 10.1038/sj.bjc.6601670

**Published:** 2004-02-24

**Authors:** A M Waterston, F Salway, E Andreakos, D M Butler, M Feldmann, R C Coombes

**Affiliations:** 1 Department of Cancer Medicine, Faculty of Medicine, Chelsea and Westminster Hospital, 369 Fulham Rd, Imperial college School of Medicine, London SW10 9NH, UK; 2 Kennedy Institute of Rheumatology Division, Faculty of Medicine, Imperial college School of Medicine, London, UK

**Keywords:** TNF, melanoma, metastasis

## Abstract

TNF is a proinflammatory cytokine involved in the pathogenesis of chronic inflammatory diseases, but also in metastasis in certain types of cancer. In terms of therapy, TNF is targeted by anti-TNF neutralising monoclonal antibodies or soluble TNF receptors. Recently, a novel strategy based on the generation of self anti-TNF antibodies (TNF autovaccination) has been developed. We have previously shown that TNF autovaccination successfully generates high anti-TNF antibody titres, blocks TNF and ameliorates collagen-induced arthritis in DBA/1 mice. In this study, we examined the ability of TNF autovaccination to generate anti-TNF antibody titres and block metastasis in the murine B16F10 melanoma model. We found that immunisation of C57BL/6 mice with TNF autovaccine produces a 100-fold antibody response to TNF compared to immunisation with phosphate-buffered saline vehicle control and significantly reduces both the number (*P*<0.01) and size of metastases (*P*<0.01) of B16F10 melanoma cells. This effect is also observed when an anti-TNF neutralising monoclonal antibody is administered, confirming the essential role TNF plays in metastasis in this model. This study suggests that TNF autovaccination is a cheaper and highly efficient alternative that can block TNF and reduce metastasis *in vivo* and trials with TNF autovaccination are already underway in patients with metastatic cancer.

TNF is an important proinflammatory cytokine involved in normal physiological immune and inflammatory processes. However, when inappropriately expressed, TNF also plays a role in the development of chronic inflammation and diseases associated with it (Andreakos *et al*, 2002). More recently, inappropriately expressed TNF was also shown to play a role in the development of cancer but in a more complex manner. Thus, in certain cancers TNF has been shown to induce haemorrhagic necrosis of tumours, whereas in others it has been shown to promote cancer (Carswell *et al*, 1975; Moore *et al*, 1999). What determines the TNF procarcinogenic or anticarcinogenic effects is not clear. It may be that this is dependent on the levels of TNF produced locally or the type of tumour and cancer involved (Balkwill, 2002).

Previous studies have suggested that one of the main mechanisms by which TNF promotes tumour growth is by upregulating metastasis. TNF activates key molecules involved in metastasis such as IL-8 (an angiogenic chemokine) and Gro*α*/KC, as well as matrix metalloproteinases (MMP) and urokinase-plasminogen activator, molecules involved in ECM degradation and cellular migration (Wu *et al*, 1999; Shin *et al*, 2000). In addition, recombinant TNF injected into mice inoculated with a methylcholanthrene-induced fibrosarcoma increased the number of lung metastases (Orosz *et al*, 1993). Similarly, tumour cell lines infected with a retrovirus carrying the TNF gene augmented metastatic activity of the tumour (Quin *et al*, 1993). In contrast, blocking TNF using the human p55-IgG fusion protein in a murine B16-BL6 melanoma model reduced the number of metastatic lung tumours temporarily over 2 weeks but not after 3 weeks, possibly due to increased immune clearance of the fusion protein (Cubillos *et al*, 1997).

Monoclonal antibodies and IgG fusion proteins are now an established approach to blocking TNF. We have previously used monoclonal antibodies against TNF to ameliorate disease in animal models of arthritis (Williams *et al*, 1992) as well as rheumatoid arthritis in the clinic (Elliot *et al*, 1994). Similarly, others have shown that administration of anti-TNF neutralising antibodies is successful in treating many other diseases such as Crohn's disease, psoriasis and spondyloarthropathies (reviewed by Andreakos *et al*, 2002). However, there are major drawbacks to the long-term use of monoclonal antibodies, murine, chimeric or human, which include a variable degree of immunogenicity and major costs, $12–15 000 p.a. (£7–9000) (Dillman *et al*, 1994; LoRusso *et al*, 1995; Philpott *et al*, 1995). TNF autovaccination is an alternative, novel approach that circumvents the problem of immunogenicity and cost of nonautologous antibodies. It is based on the generation of autologous human autoantibodies by immunisation with a self-antigen to which a T-cell epitope has been added (Dalum *et al*, 1997). Autoantibodies against TNF are raised by immunisation with recombinant TNF protein containing inserted hen egg lysozyme (HEL) or ova albumin (OVA) epitopes. This leads to a T helper response specific for the foreign parts of the recombinant molecule and allows the generation of antibodies against TNF that neutralise and eliminate it (Dalum *et al*, 1999). When we previously used TNF autovaccination in a murine collagen-induced arthritis model, we found that this resulted in the generation of high titres of anti-TNF antibodies that ameliorated the disease and decreased the severity of arthritis (Dalum *et al*, 1999).

In this study, we examined whether blockade of TNF could reduce metastasis in the murine B16F10 melanoma model (Poste *et al*, 1980) and compared the efficacy of anti-TNF monoclonal antibodies and the TNF autovaccination technology in reducing metastasis. Our results indicate that both the conventional anti-TNF monoclonal antibody treatment and TNF autovaccination are successful in reducing the number and size of lung metastases in this model and supports the use of these agents in clinical trials in patients with metastatic cancer.

## MATERIALS AND METHODS

### Immunisation with TNF autovaccination

**Figure 1:**
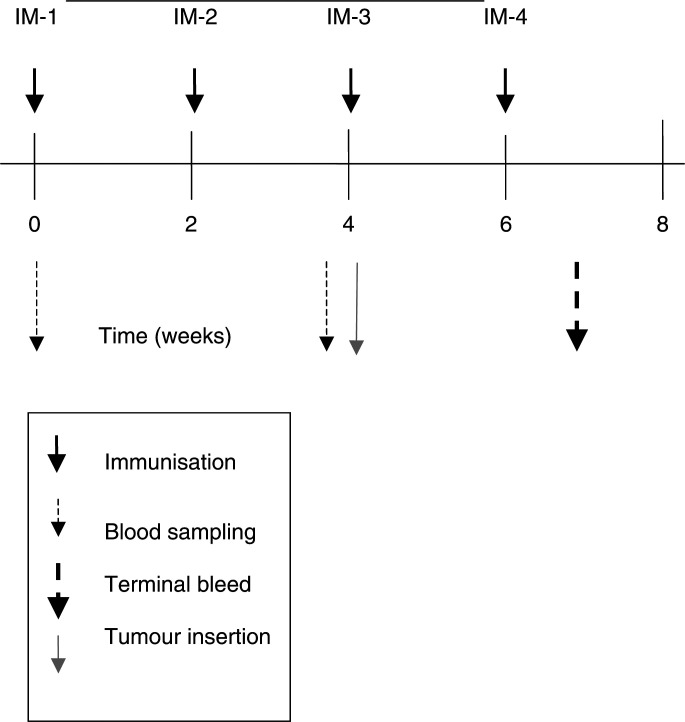
Experimental design and study calendar showing immunisation protocol and tumour insertion. The diagram shows the time points for immunisation of TNF autovaccine, blood sampling, injection of the B16F10 cells (tumour insertion) and termination of the experiment at the terminal bleed.

The immunisation regime was first developed in 4- to 5-week-old male DBA/1 mice as described in our previous study in collagen-induced arthritis (Dalum *et al*, 1999). C57BL/6 mice (4-week old) (12/group) were immunised intradermally at the base of the tail with 100 *μ*g of TNF autovaccine in phosphate-buffered saline (PBS) emulsified with complete Freund's adjuvant (CFA), in a volume of 100 *μ*l (Figure 1

). Three subsequent boosts with TNF autovaccine in incomplete Freund's adjuvant (IFA) were performed at two-weekly intervals. In the immunisation study, the control used was HEL in CFA and three boosters in IFA and the blood samples were taken prior to study commencement and at two-weekly intervals thereafter, for 12 weeks. For the B16F10 metastasis model, mice were immunised with PBS in CFA/IFA as a control. After 3 weeks, the mice were killed, lung tumours counted, and the diameter measured in two perpendicular planes. To reduce biased selection in the B16F10 melanoma metastasis model, the treatments were blinded coding them A and B prior to injection and the codes broken at the end of the experiment after the data were collected and analysed.

Blood samples were taken prior to immunisation and prior to tumour insertion. On killing the animals at the end of the experiments, a terminal bleed was also undertaken. The blood was allowed to clot and then centrifuged at 12 000 r.p.m. for 5 min and the top layer of sera removed. The serum was stored at −20°C prior to use in immunological assays to determine antibody levels. The experiments were carried out according to Protocol 8 on License PPL 70 3831, issued by The Home Office Animal Procedures Section, London and adhered to the UKCCR guidelines (Workman *et al*, 1998).

### Treatment with Anti-TNF monoclonal antibodies

C57BL/6 mice (6-week old) were injected intraperitoneally (i.p.) with 500 *μ*g 100 *μ*l^−1^ of anti-TNF monoclonal antibodies (CV1q) three times per week or PBS. Again to reduce biased selection, the treatments were blinded coding them A and B prior to insertion into the mice and the code was broken after the data were collected and analysed. The monoclonal antibodies were a rat mouse fusion protein (gift from Dr DB Scallon, Centocor, Malvern, USA). The day after the initial injection, 10^5^ B16F10 murine melanoma cells in 200 *μ*l of PBS were injected into the tail vein of each mouse. After 3 weeks, the mice were killed and the lung tumours counted and the diameter measured as described above.

### Antibody detection assays

Microtitre plates were coated with 1 *μ*g ml^−1^ of TNF in PBS at 100 *μ*l well^−1^ and incubated overnight at 4°C. The plates were blocked with 2% bovine serum albumin (BSA) in PBS (200 *μ*l well^−1^) for 1 h at 25°C and washed with 0.5% Tween in PBS after this and all subsequent steps. A measure of 100 *μ*l of one in three serial dilutions of serum samples and the positive and negative control sera were incubated for 1 h at 25°C. The detection antibody (100 *μ*l well^−1^) was diluted in 0.5% BSA in PBS and incubated for 1 h at 25°C. A sheep anti-mouse polyclonal detection antibody conjugated to horseradish peroxidase was used. These samples were developed with a peroxidase substrate system TMB, the reaction was stopped with 1 M H_2_SO_4_ and absorbance read at 450 nm. Since standard known amounts of antibodies to these antigens were unavailable, eight serial dilutions of sample sera were made and their titres taken as the dilution that gave an OD corresponding to that of the negative control sera. The negative control sera were from unimmunised mice and the positive control sera were pooled sera from previously immunised mice that had been tested by ELISA and found to have antibodies that bound TNF.

### Cell culture and detection of KC

B16F10 murine melanoma cells were grown at 37°C in 5% CO_2_ in RPMI supplemented with 10% foetal bovine serum (BioWhittaker), penicillin (100 U ml^−1^) and streptomycin (100 *μ*g ml^−1^). B16F10 cells were plated in 96-well plates at 2 × 10^6^ cells ml^−1^ and 200 *μ*l well^−1^. The cells were stimulated with LPS at 10 *μ*g ml^−1^; some cells were further stimulated with murine TNF (mTNF) at 10 or 100 ng ml^−1^ and other cells with media as a control. The plates were incubated 37°C. Supernatants were removed at 24 and 72 h and stored at −20°C for immunological detection assays. KC was measured by ELISA as described previously (Sarawar *et al*, 2002). Detection range for the KC was 10 000–4.5 ng ml^−1^.

## RESULTS

### TNF autovaccination induces high levels of anti-TNF autoantibody production

**Figure 2:**
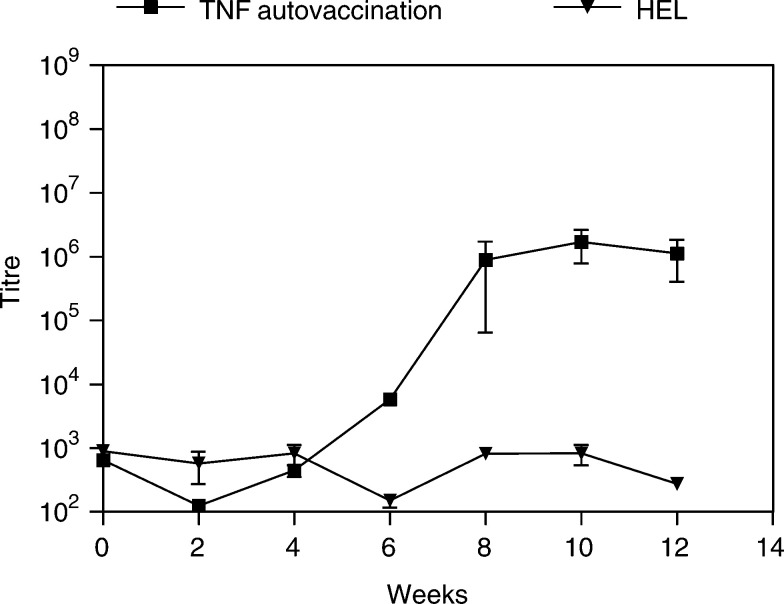
Anti-TNF antibody response in mice immunised with TNF autovaccine. Two groups of 4-week-old C57BL/6 mice (*n*=8) were initially immunised with 100 *μ*g TNF autovaccine or HEL in CFA then, at two-weekly intervals, boosted three times with 100 *μ*g of the same antigen in IFA. Serum samples were taken prior to immunisation and every 2 weeks thereafter. The TNF antibody levels were measured in an ELISA. The titre values were calculated as the dilution of serum, which corresponded to titres from nonimmunised mice. The mean levels±s.e.m. are shown. This is representative of two independent experiments.

We investigated the ability of the novel TNF autovaccination to induce high anti-TNF antibody titres. We immunised C57BL/6 mice with the TNF autovaccine in the presence of CFA/IFA and compared the antibody responses to mice immunised with HEL in CFA/IFA. We found that in the TNF autovaccination group, the anti-TNF antibody titres started rising at week 4 peaked at week 8 and reached plateau levels thereafter (Figure 2

). This was compared to the HEL control group that had no anti-TNF antibody response. This was similar to the antibody response seen in BALB/c and CH3 mice (Dalum *et al*, 1999), but different from the biphasic antibody response seen at 6 and 10 weeks in DBA/1 mice after immunisation with TNF autovaccine (data not shown).

### Anti-TNF monoclonal antibodies on lung metastases in the B16F10 melanoma model

**Figure 3:**
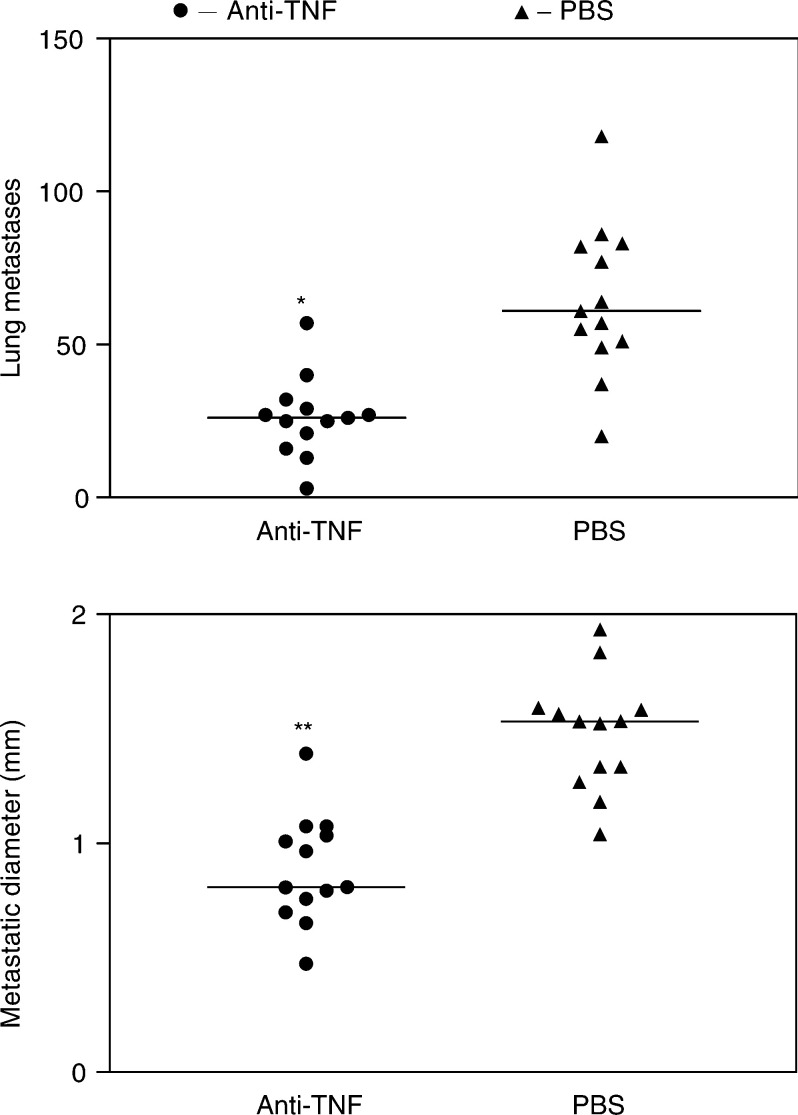
Effect of anti-TNF monoclonal antibody treatment in the B16F10 metastatic model. C57BL/6 mice (8-week old) (anti-TNF group) were treated with i.p. injections of anti-TNF monoclonal antibodies (CV1q) 500 *μ*g 100 *μ*l^−1^ mouse^−1^. The PBS group were treated with 100 *μ*l PBS i.p. All mice were treated three times per week. Following their first injection, the mice were injected i.v. with 10^5^ B16F10 cells. The mice were killed 3 weeks later, the lung metastases counted and mean diameters measured with callipers. The median is shown and the Kruskal–Wallis and Dunn's multiple comparison nonparametric test calculates significance, ^*^P<0.01 and ^**^P<0.001.

First, to confirm the role of TNF in metastases in the B16F10 melanoma model, we used an anti-TNF neutralising antibody, CV1q. Mice were treated i.p. with either PBS or anti-TNF monoclonal antibodies (CV1q), and then B16F10 melanoma cells were injected into the tail vein. We found that mice treated with anti-TNF monoclonal antibodies had significantly less and smaller metastases compared to the mice treated with PBS (Figure 3A and B

).

### TNF blockade through immunisation using a novel TNF autovaccine on lung metastases *in vivo* in the B16F10 melanoma model

Having shown that blocking TNF can have an effect on metastasis, we examined the effect of immunisation with TNF autovaccine in the B16F10 melanoma model in three independent experiments. Mice were immunised with TNF autovaccine or PBS in CFA and boosted with IFA. The B16F10 melanoma cells were injected into the tail vein of each mouse 4 weeks after their first immunisation and the tumours were allowed to grow for 3 weeks.

**Figure 4:**
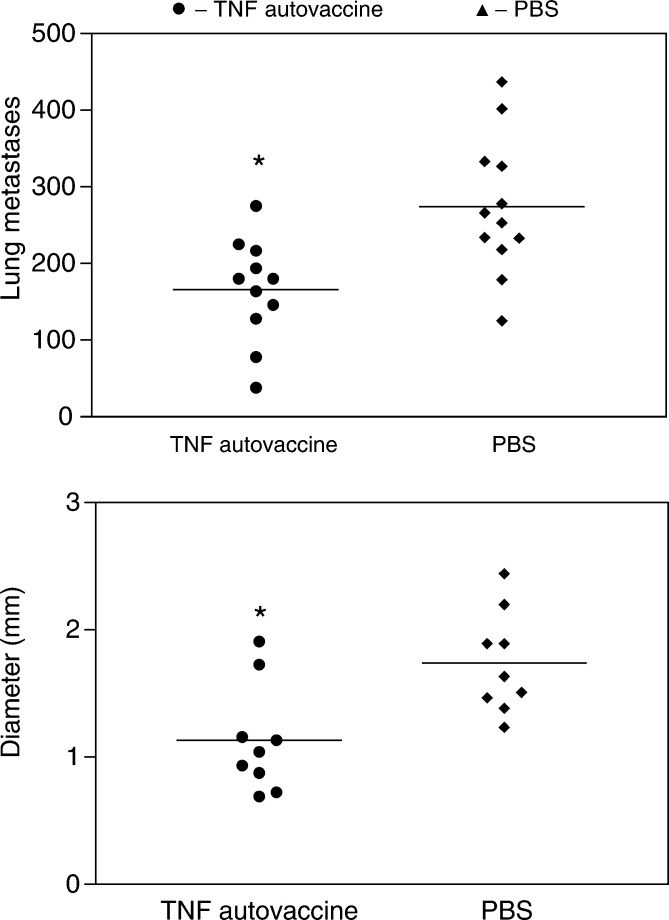
Lung metastases in mice immunised with TNF autovaccine in the B16F10 melanoma model. C57BL/6 mice (4-week old) (*n*=12/treatment group) were immunised i.m. with 100 *μ*g TNF autovaccine or PBS in CFA and boosted three times at two-weekly intervals in IFA. B16F10 cells (10^5^) in 200 *μ*l of PBS were injected into their tail veins at 4 weeks post initial immunisation. At 7 weeks, the mice were killed and the lungs excised. The numbers of lung metastases were counted (**A**) and six randomly selected tumours measured with calipers (**B**). Each group was compared to the PBS control and the one-Way ANOVA test was used to evaluate results. The mean and *P*-values for each group are shown. A representative of three independent experiments is shown, ^*^P<0.01.

**Figure 5:**
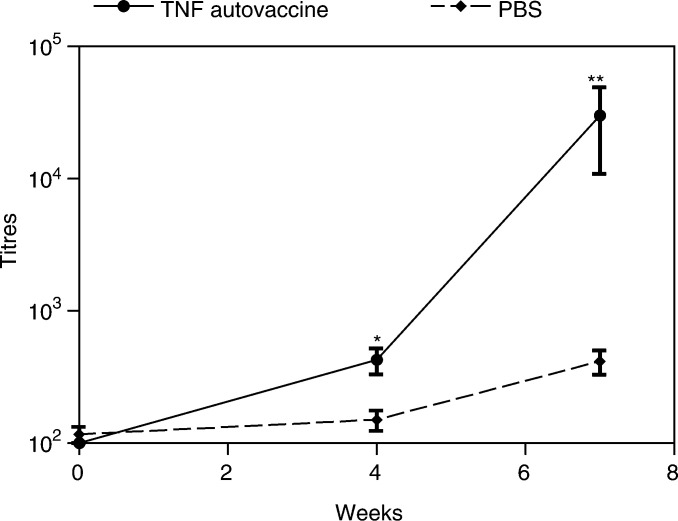
Anti-TNF antibody titres in mice immunised with TNF autovaccination in the B16F10 metastatic model. C57BL/6 mice (4-week old) (*n*=12/treatment group) were immunised and tumour cells inserted as described in Figure 3. Mice were bled at 0.4 and 7 weeks. The TNF antibody levels were measured in an ELISA. The titre values were calculated as the dilution, which gave an OD equal to nonimmunised mice. Mean and s.e.m. for each group of mice are shown and the results were evaluated using one-way ANOVA comparing each group to the PBS control, ^*^P<0.05 and ^**^P<0.001.

We found that the group of mice immunised with the TNF autovaccine showed significant reduction in metastatic lesions compared with those treated with PBS (Figure 4A

). The sizes of the metastases were also significantly reduced in the mice immunised with TNF autovaccine compared to the PBS control group (see Figure 4B). The reason for not using HEL with CFA as the control was that in previous experiments it had led to increased murine morbidity. The morbidity may have been due to the use of a strong antigen such as HEL with CFA inducing ulcerations on the surface of the animals; this was not seen with TNF autovaccination. Upon killing the animals, a terminal bleed was carried out. In these experiments, blood was also sampled from mice prior to immunisation and prior to tumour implantation. The anti-TNF antibodies were then measured in the sera using an ELISA-based detection system (Figure 5

). We found that the anti-TNF titres in the TNF autovaccination group increased dramatically by the end of the experiment unlike the PBS control group.

### TNF synergises with other stimulants to induce KC production in B16F10 cells

**Figure 6:**
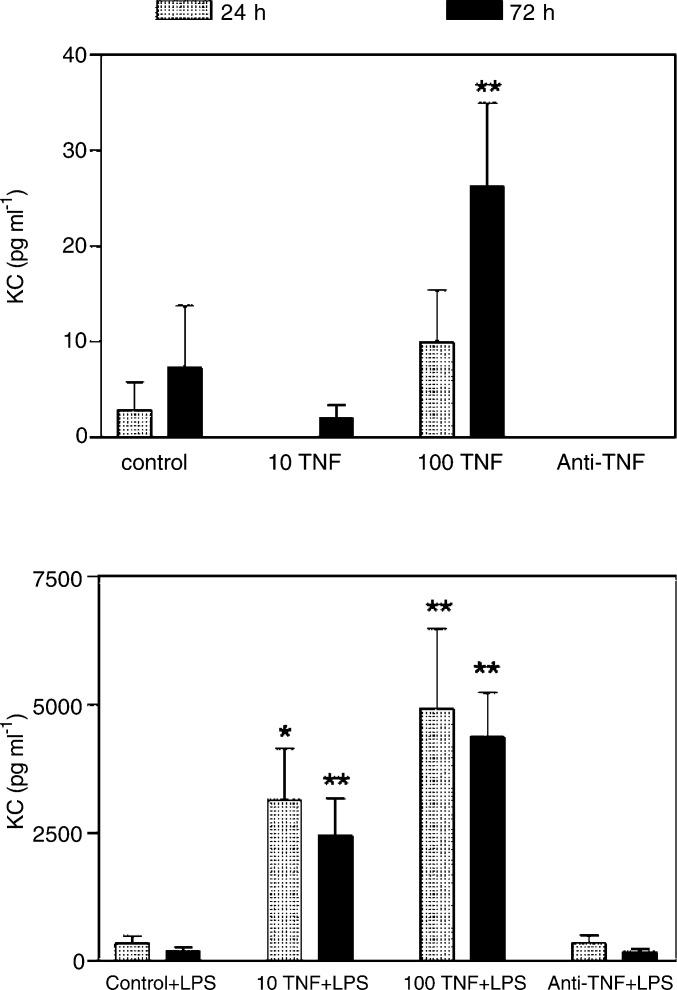
Effects of TNF and LPS on KC production in B16F10 cells. Cells were plated out in triplicate at 2 × 10^6^ *μ*l ml^−1^ in 200 *μ*l well^−1^. To each well was added 10 *μ*g ml^−1^ LPS, 10 or 100 ng ml^−1^ mTNF, 10 *μ*g ml^−1^ murine anti-TNF monoclonal antibodies or media as a control as shown in the legend. Supernatants were removed at 24 and 72 h to measure KC levels by ELISA. Statistical significance was measured by the one-way ANOVA test. These are pooled results from two experiments.

In conjunction with the *in vivo* experiments, the ability of mouse recombinant TNF to induce the expression of cytokines/chemokines such as IL-6, IL-1, VEGF, GMCSF and KC, and also TNF was examined as this could account for some of the metastatic effects of TNF. However, we found no detectable IL-6, IL-1, VEGF, GMCSF or endogenous TNF when B16F10 cells were left unstimulated or stimulated with TNF and or LPS *in vitro*, *in vivo* this may differ. In contrast, the chemokine KC (the murine equivalent of human MGSA/Gro*α*), previously described to play a role in metastasis (Smith *et al*, 1998), could be detected by ELISA when B16F10 cells were cultured for 24 and 72 h *in vitro* with either TNF or anti-TNF neutralising monoclonal antibodies. TNF increased KC production above background levels after 72 h when 100 *μ*g ml^−1^ of TNF was added to B16F10 cultures. Anti-TNF neutralising antibodies added to B16F10 cells did not reduce KC (Figure 6

). Interestingly, we found that under certain circumstances TNF can induce KC expression at 24 and 72 h. Thus, by adding 10 *μ*g ml^−1^ LPS at the onset of the culture, the production of KC was increased in B16F10 cells both at 24 and 72 h (Figure 6). The addition of TNF in these cultures greatly upregulated KC production in a dose-dependent manner. This suggested that in combination with certain stimuli, TNF might have a synergistic effect in inducing KC in B16F10 cells. Surprisingly, anti-TNF had no effect in the LPS-induced KC production in these cultures, suggesting that LPS did not increase endogenous TNF production in the B16F10 cells.

## DISCUSSION

The B16F10 murine melanoma model has been used by a number of groups to examine the process of metastasis (Poste *et al*, 1980). It offers the advantage of injecting B16F10 melanoma cells into mice, which can metastasise to the lungs. Tumours in the lungs are then readily visible as B16F10 cells are pigmented.

In this study, we used the model to investigate the role of TNF in promoting metastasis and the ability of the TNF autovaccination technology to generate high titre anti-TNF autoantibodies to inhibit TNF function *in vitro*. We have also used TNF autovaccination to generate high titres of anti-TNF antibodies *in vivo* and ameliorate collagen-induced arthritis in DBA/1 mice (Dalum *et al*, 1999). We found that in C57BL/6 mice, TNF autovaccination also induced high levels of anti-TNF antibodies in the serum that plateaued 8 weeks after immunisation (Figure 2). This was similar to the anti-TNF antibody titres induced by TNF autovaccination in BALB/c and CH3 mice (Dalum *et al*, 1999), but different from the biphasic antibody response seen in DBA/1 mice after immunisation with TNF autovaccination. The reason for this is not clear, but may simply be due to differences in the binding and presentation of epitopes between the distinct MHC class II haplotypes. Alternatively, this may be due to the different types of assay used to detect anti-TNF antibodies. In DBA/1 mice, only biologically active anti-TNF antibodies were measured using a bioassay that quantifies their ability to inhibit TNF cytotoxicity, whereas in BALB/c and CH3 mice a biochemical receptor assay was used and for the C57BL/6 mice all anti-TNF antibodies were measured using an ELISA assay. In terms of clinical trials of TNF autovaccination in patients, the potential variability in antibody responses will be undesirable as it may result in variability in efficacy or make interpretation of data difficult. However, this problem should we hope be circumvented by using promiscuously binding T helper epitopes in the recombinant vaccines.

The induction of high levels of anti-TNF antibodies reduced the number of metastases reaching and establishing themselves in the lungs as well as their size in the B16F10 murine melanoma model when compared to mice immunised with PBS in CFA. Prior to this, we ensured that blocking TNF by a known mechanism, monoclonal antibodies to TNF, could also reduce tumour metastasis. For this, we examined the effect of treating mice with anti-TNF monoclonal antibodies upon tumour insertion and had a reduction in the lung metastases compared to the PBS-treated control group. Further support for this comes from findings of Cubillos *et al* (1997), who used a human TNF receptor fusion protein in a B16-BL6 melanoma model. Although it was found that the human soluble TNF fusion protein significantly inhibited lung metastases, this effect was short lived possibly due to the immunogenicity of the human protein increasing its clearance. In our system, the TNF monoclonal antibody, CV1q, is a rat/mouse fusion protein, and as the two species share more similarity, the rat portion does not produce as strong an anti-rat immune response when injected into mice. TNF autovaccination generated autologous antibodies and therefore the antibodies would not have been recognised and cleared by the mouse immune response. Both the autoantibodies generated by immunisation with TNF autovaccine and the anti-TNF monoclonal antibodies reduced the levels of metastases measured after 3 weeks, which shows that their effect lasted longer than that seen with the human TNF receptor fusion protein (Cubillos *et al*, 1997). The optimal time for tumour insertion was based on the previous data by Dalum *et al* (1999) and on the immunisation of C57BL/6 mice (Figure 2). These data showed that the anti-TNF antibodies measured in mice treated with TNF autovaccination were present from 4 to 10 weeks. Others have shown a subsequent decline in anti-TNF antibody levels at 3–6 months (data not shown). Consequently, the time for tumour insertion was chosen as 4 weeks to correspond to the rise in antibody levels as seen in Figure 5 in which the anti-TNF antibodies were measured in mice with melanoma. Unfortunately, the examination of the TNF autovaccine in established tumours in the B16F10 melanoma model was not possible because of two reasons. First, the B16F10 melanoma model is an acute rapidly progressing model that develops metastases within 2–3 weeks. As TNF autovaccination requires 4–6 weeks to induce optimal TNF autoantibodies, the tumour burden by that time would have been too great, with many mice already dead or in great pain for the procedure to be ethically acceptable. Second, most of the TNF tumour-promoting effects that have been described concern processes that occur during metastasis. Therefore, our main purpose was to investigate if blocking TNF by TNF autovaccination had an effect on the establishment of metastasis from cells in circulation.

We then investigated potential mechanisms involved in the reduction of metastases seen with TNF autovaccination. Owing to the small amounts of serum obtained from each mouse, we were unable to perform a range of cytokine evaluations. The only inflammatory marker measured was serum amyloid P, which showed no difference in levels between the treated groups (data not shown). As we were unable to examine any cytokines in the mice sera, we examined the effects of blocking TNF *in vitro*. We found that at high doses of TNF or in the presence of LPS, TNF upregulates KC in B16F10 cells. Interestingly, studies of mice that had their primary tumours removed surgically and were then injected with LPS or saline had increased lung metastases in the LPS injected group, indicating that LPS may activate mechanisms involved in the metastatic processes (Pidgeon *et al*, 1999). Furthermore, *in vivo* and *in vitro* studies have shown that LPS can increase vascular permeability, tumour invasion as well as increase inducible nitric oxide synthase and MMP2 production, two factors known to play a role in metastasis (Harmey *et al*, 2002). It may be that *in vivo* LPS or LPS-like pathways also upregulate KC, although these groups did not measure this. Although LPS may not be physiologically relevant in this context, our study still demonstrates that under certain conditions that may be met *in vivo*, TNF can upregulate KC production. Certainly, in humans TNF alone is able to upregulate Gro*α* (the human equivalent of KC) in keratinocytes (Li and Thornhill, 2000), fibroblasts and human melanoma cell lines (Shattuck *et al*, 1994). Interestingly, KC is a proinflammatory chemokine that has been found to play a key role in inducing neovascularisation of corneal epithelial cells (Strieter *et al*, 1995) and in promoting metastasis in squamous carcinoma cells (Smith *et al*, 1998). Thus, part of the effect of blocking TNF by TNF autovaccination may be due to a decrease in the production of KC. Decreased KC production, in particular, may result in the reduced ability of B16F10 cells to invade lung tissue and also reduce neovascularisation of tumours in the lung, consequently decreasing the size and numbers of metastatic lesions. In our cell system, however, we were unable to demonstrate any reduction in KC when anti-TNF was added to B16F10 cells, which maybe due to insufficient TNF production by B16F10 cells despite LPS stimulation. Therefore, we can only speculate that KC maybe one of the mechanisms by which blocking TNF reduces metastasis in our *in vivo* melanoma model. TNF may also directly affect neovascularisation and angiogenesis by upregulating factors such as VEGF as previously shown in human macrophage cells and synovial cells from rheumatoid arthritis patients (Paleolog *et al*, 1998; Kiriakidis *et al*, 2003). *In vivo*, some cancers particularly epithelial tumours produce TNF; however, in other cancers, stromal cells are a source of TNF that can then have an effect on the tumours (Balkwill, 2002).

Overall, our study shows that although metastasis is a complex multisystem process involving a large number of different molecules, in the B16F10 murine melanoma model TNF plays an important and rate-limiting role. This is likely to be partly due to its ability to increase the expression of prometastatic molecules such as KC. In addition, our study demonstrates that the TNF autovaccination technology is a very efficient strategy in producing anti-TNF antibodies in mice and preventing in that way the number and size of metastases. This strategy is also safe in this experimental system as no adverse effects or increase in tumour growth or metastasis was seen. We are currently conducting a phase I human clinical trial to evaluate the effect of blocking TNF by using TNF autovaccination in patients with a variety of cancers. Results from these trials are eagerly awaited. If this strategy is found to be effective in human trials, it would be a cost-effective alternative to infusing monoclonal antibodies and more convenient for patients who would possibly only require three injections for a 3-month benefit.
